# Antimicrobial resistance in *Streptococcus pneumoniae*: a retrospective analysis of emerging trends in the United Arab Emirates from 2010 to 2021

**DOI:** 10.3389/fpubh.2023.1244357

**Published:** 2023-11-23

**Authors:** Abiola Senok, Jens Thomsen, Najiba M. Abdulrazzaq, The UAE AMR Surveillance Consortium, Godfred Antony Menezes, Carole Ayoub Moubareck, Dean Everett

**Affiliations:** ^1^College of Medicine, Mohammed Bin Rashid University of Medicine and Health Sciences, Dubai, United Arab Emirates; ^2^School of Dentistry, Cardiff University, Cardiff, United Kingdom; ^3^Abu Dhabi Public Health Center, Abu Dhabi, United Arab Emirates; ^4^Department of Pathology and Infectious Diseases, Khalifa University, Abu Dhabi, United Arab Emirates; ^5^Al Kuwait Hospital Dubai, Emirates Health Establishment, Dubai, United Arab Emirates; ^6^Public Health Sector, Ministry of Health and Prevention, Dubai, United Arab Emirates; ^7^Department of Medical Microbiology and Immunology, RAK Medical and Health Sciences University, Ras Al-Khaimah, United Arab Emirates; ^8^College of Natural and Health Sciences, Zayed University, Dubai, United Arab Emirates; ^9^Biotechnology Center, Khalifa University, Abu Dhabi, United Arab Emirates; ^10^Infection Research Unit, Khalifa University, Abu Dhabi, United Arab Emirates

**Keywords:** *Streptococcus pneumoniae*, pneumococcal conjugate vaccine, serotyping, invasive pneumococcal disease, antimicrobial resistance

## Abstract

**Introduction:**

Although pneumococcal conjugate vaccines (PCV) have been effective in reducing the burden of *Streptococcus pneumoniae* infections, there is a paucity of data on the relationship with antimicrobial resistance (AMR) trends in the Arabian Gulf region. This study was carried out to assess *S. pneumoniae* resistance trends in the United Arab Emirates (UAE) where PCV-13 vaccination was introduced in 2011.

**Methods:**

Retrospective analysis of *S. pneumoniae* demographic and microbiological data collected as part of the national AMR surveillance program from 2010 to 2021 was carried out. A survey of reporting sites and hand searching of annual reports of local health authorities was carried out to identify data on *S. pneumoniae* serotypes as this is not included in the AMR surveillance database.

**Results:**

From 2010 to 2021, 11,242 non-duplicate *S. pneumoniae* isolates were reported, increasing from 324 in 2010 to 1,115 in 2021. Factoring in annual increment in the number of surveillance sites, the number of isolates per site showed an upward trajectory from 2015 to 2018 and declined in 2020 with the onset of the pandemic. The majority of isolates (*n/N* = 5,751/11,242; 51.2%) were from respiratory tract specimens with 44.5% (*n/N* = 2,557/5,751) being nasal colonizers. Up to 11.9% (*n/N* = 1,337/11,242) were invasive pneumococcal disease (IPD) isolates obtained from sterile site specimens including blood (*n* = 1,262), cerebrospinal (*n* = 52), pleural (*n* = 19) and joint (*n* = 4) fluid; and were predominantly from pediatric patients. The downward trend for amoxicillin and for penicillin G at the non-meningitis and meningitis as well as oral penicillin breakpoints was statistically significant. In contrast, increasing trends of resistance were seen for levofloxacin, moxifloxacin, trimethoprim/sulfamethoxazole and erythromycin. IPD and non-IPD isolates showed similar demographic and AMR trends. None of the surveillance sites carried out *S. pneumoniae* serotyping and handsearching of annual reports did not yield this information.

**Conclusion:**

The increasing trend of pneumococcal disease and AMR with emergence of isolates with MDR phenotype despite is of concern. In the absence of *S. pneumoniae* serotyping the role of non-vaccine serotypes in driving this pattern remains unknown. There is an urgent need for serotype, genomic and AMR surveillance of *S. pneumoniae* isolates in the UAE.

## 1 Introduction

*Streptococcus pneumoniae* is a major cause of disease in children and adults with associated high burden of morbidity and mortality. In 2015, the global mortality rate attributed to pneumococcal infection was 45 deaths (29-56) per 100,000 among children aged 1–59 months (1). The spectrum of clinical manifestations ranges from non-invasive disease such as otitis media, to invasive pneumococcal disease (IPD) such as meningitis and bacteremia. In a recent report on the global analysis of lower respiratory tract infections, pneumococcal pneumonia caused more deaths than all other etiologies combined accounting for 1,189,937 deaths in 2016 (2). Although there are >100 serotypes of *S. pneumoniae* (3, 4), only a limited number are responsible for most IPD. The introduction of vaccines played a role in reducing the burden of morbidity and mortality associated with common vaccine preventable infectious diseases (5, 6). Indeed, this has been demonstrable with pneumococcal conjugate vaccines (PCV), wherein the initial introduction of the seven-valent pneumococcal conjugate vaccine (PCV7) and the subsequent 13-valent vaccine (PCV13) have been effective in reducing the burden of pneumococcal disease in children and adults (7).

Antimicrobial resistance (AMR) is a major global health threat with ~700,000 attributable deaths annually and a projected increase to 10 million by 2050 (8). It has been suggested that by reducing the numbers of the target microbe (both antibiotic susceptible and resistant strains) in circulation, vaccination programs could be a promising additional weapon in the fight against AMR (6, 9). Furthermore, with fewer occurrences of clinical infections following vaccination, a drop in antibiotic utilization is expected which could reduce selection pressure and emergence of resistance strains. For *S. pneumoniae*, pneumococcal carriage has been described as a critical source of horizontal spread in the community and the effect of vaccination on reduction of nasopharyngeal colonization could also impact antibiotic resistance (10, 11). However, the occurrence of serotype replacement by non-vaccine serotypes and the association with antibiotic resistant pneumococcal strains could negate the expected reduction in AMR trends (7, 9, 10, 12). This highlights the need for surveillance of AMR in *S. pneumoniae* particularly the tracking of emergent trends after the onset of a PCV vaccination program.

The UAE is highly cosmopolitan with dynamic population movement of large numbers of expatriate residents and tourists from across the world. Hence the emergence and dissemination of AMR pathogens is a concern particularly with reports of novel and variant strains in circulation (13, 14). In the 2011–2013 Survey of Antibiotic Resistance (SOAR) in the Gulf States, susceptibility of *S. pneumoniae* to most of the antibiotics tested was found to be consistently lower in UAE compared to other countries in the region (15). An earlier report by Senok et al. (16) had identified a high level of penicillin resistance, elevated macrolide and fluoroquinolone resistance and the occurrence of multidrug resistance phenotypes among *S. pneumoniae* isolated between 2004 and 2006 from patients with community acquired respiratory tract infections in the UAE. In addition, despite the significant risk factors for pneumococcal disease in the Arabian Gulf region, and the calls for heightened pneumococcal surveillance, there remains a paucity of published literature on the burden of pneumococcal infections (17). Indeed, in the UAE, the only two published studies on pneumococcal burden are based on single center data obtained prior to the 2007 introduction of PCV-7 (18, 19). These studies showed a higher incidence rate of pneumococcal disease relative to developed countries (19) with *S. pneumoniae* as causative agent of 9% of community acquired pneumonia (18). To address this gap in the literature, this report describes *S. pneumoniae* epidemiology and antibiotic resistance trends in the UAE over a twelve-year period.

## 2 Methods

This study is a retrospective data analysis for the twelve-year period 2010–2021. This timeframe includes one year prior to the 2011 introduction of PCV-13 in the UAE. AMR trends in *S. pneumoniae* were assessed by analysis of routine patient care national level AMR surveillance data.

### 2.1 Data collection

The national AMR surveillance data is collected from a network of participating healthcare facilities and diagnostic laboratories across the country. These include primary, secondary and tertiary care facilities across governmental and private healthcare sectors. All data are collected from routine patient care, cleaned, and analyzed using a unified platform^1^ as described by Thomsen et al. (20). Training on data collection is provided to ensure quality assurance, standardization and accuracy. The fully anonymized data includes demographic data (age, gender, nationality, hospital site/location etc.), clinical and microbiological data such as specimen source and antibiogram. Pediatric age group was defined as newborn up to 18 years and those aged 19 years and above were categorized as adults. As clinical diagnosis is not routinely included in the dataset, the isolation of *S. pneumoniae* from blood, cerebrospinal fluid and other normally sterile body sites (e.g., joint, pleural and pericardial fluid) were used as indicators of IPD in line with the Centers for Disease Control and Prevention IPD case definition (https://ndc.services.cdc.gov/case-definitions/invasive-pneumococcal-disease-2017/).

### 2.2 Bacterial identification and antimicrobial susceptibility testing

The participating centers used at least one commercial, automated system for bacterial identification and antimicrobial susceptibility testing. These automated systems include VITEK^®^ (BioMérieux SA, Craponne, France), BD Phoenix™ (Becton Dickinson, New Jersey, USA) and MicroScan WalkAway (Beckman Coulter, Brea, CA, USA) and were used in conformity with manufacturer guidelines. Only one laboratory relied solely on manual system for bacterial identification using API^®^ (Analytical Profile Index. BioMérieux SA, Craponne, France). Two laboratories used manual antimicrobial testing methods (disc diffusion/Kirby Bauer). For the reporting of antimicrobial resistance, CLSI breakpoints were routinely applied by reporting sites and at the central level to determine susceptibility profiles of isolates (21).

### 2.3 *S. pneumoniae* serotype distribution

Having an understanding of the *S. pneumoniae* serotype in circulation is of tremendous importance. However, the national AMR surveillance dataset does not include the crucial information on *S. pneumoniae* serotypes. To determine if *S. pneumoniae* serotyping was being carried out and if so to obtain data on the serotypes that have identified, we used two approaches to source for this data. Firstly, participating sites and laboratories in the national AMR surveillance program were requested via email questionnaire to indicate if *S. pneumoniae* serotyping is currently being undertaken or had previously been carried out in the last ten years and if so, they were requested to provide the data if available. Secondly, handsearching of publicly available annual reports (for the years 2010–2020) of the health authorities namely Department of Health, Abu Dhabi (DOH), Dubai Health Authority (DHA), and the Ministry of Health and Prevention (MoHAP) for reported data on *S. pneumoniae* serotypes and IPD was carried out. The DOH Quarterly Communicable Disease Bulletins (https://www.doh.gov.ae/en/resources/publication) and Open Data Dashboard (https://www.doh.gov.ae/en/resources/opendata), the DHA statistical report (https://www.dha.gov.ae/en/open-data) and MOHAP Opendata dashboard (https://mohap.gov.ae/en/open-data/mohap-open-data) were searched.

### 2.4 Statistical analysis

Data analysis was routinely carried out using the WHONET 2023 software. For additional statistical analysis other software packages used were IBM SPSS Statistics, version 28.0 (IBM SPSS Software), and EpiInfo^TM^ for Windows v7.2.4.2022, Centers for Disease Control and Prevention. Statistical significance of temporal trends for antimicrobial resistance was calculated if data from at least five consecutive years was available. Statistical significance of trends is expressed as a *p*-value, calculated by a Chi-square for trend test (extended Mantel-Haenszel). A *p*-value of <0.05 was considered statistically significant. A 95% confidence interval is determined for the percentage of resistance and susceptibility based on the Wilson Score Interval with or without continuity correction method for calculating confidence intervals for a sample proportion (normal approximation to a binomial distribution) (22).

## 3 Results

### 3.1 Distribution of reporting sites for national AMR surveillance

**Figure 1:**
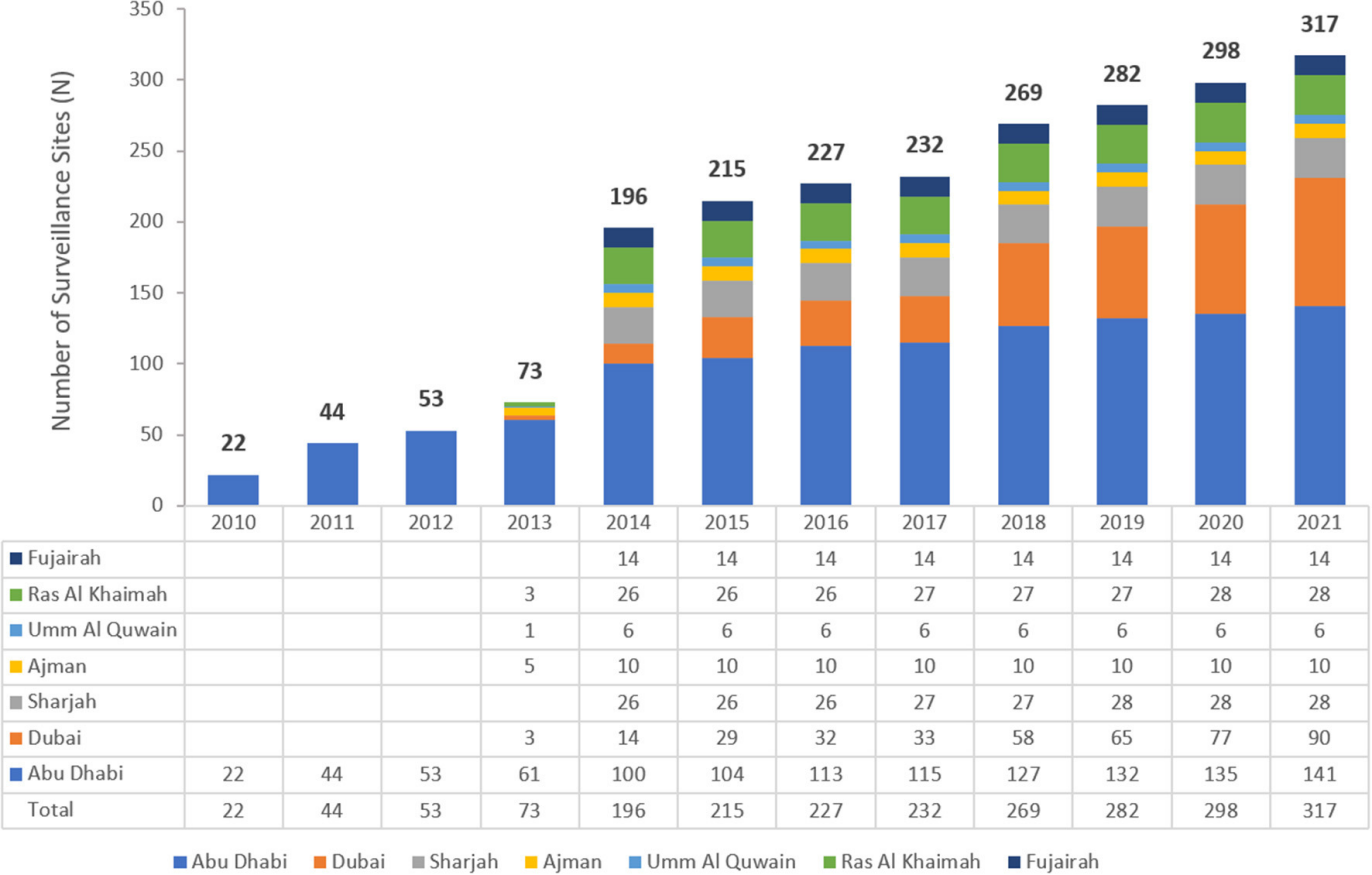
Number of surveillance sites per year and Emirate (2010–2021).

The number of reporting sites increased from 22 in 2010 to 317 in 2021 (Figure 1). These comprised of primary, secondary and tertiary care facilities across the public and private health sectors. From 2014 to 2021, the participating centers were distributed across all the seven emirates in the country in contrast to the period 2010–2012 when data was only obtained from Abu Dhabi emirate and from five emirates in 2013.

### 3.2 Bacterial population and demographic distribution

**Figure 2:**
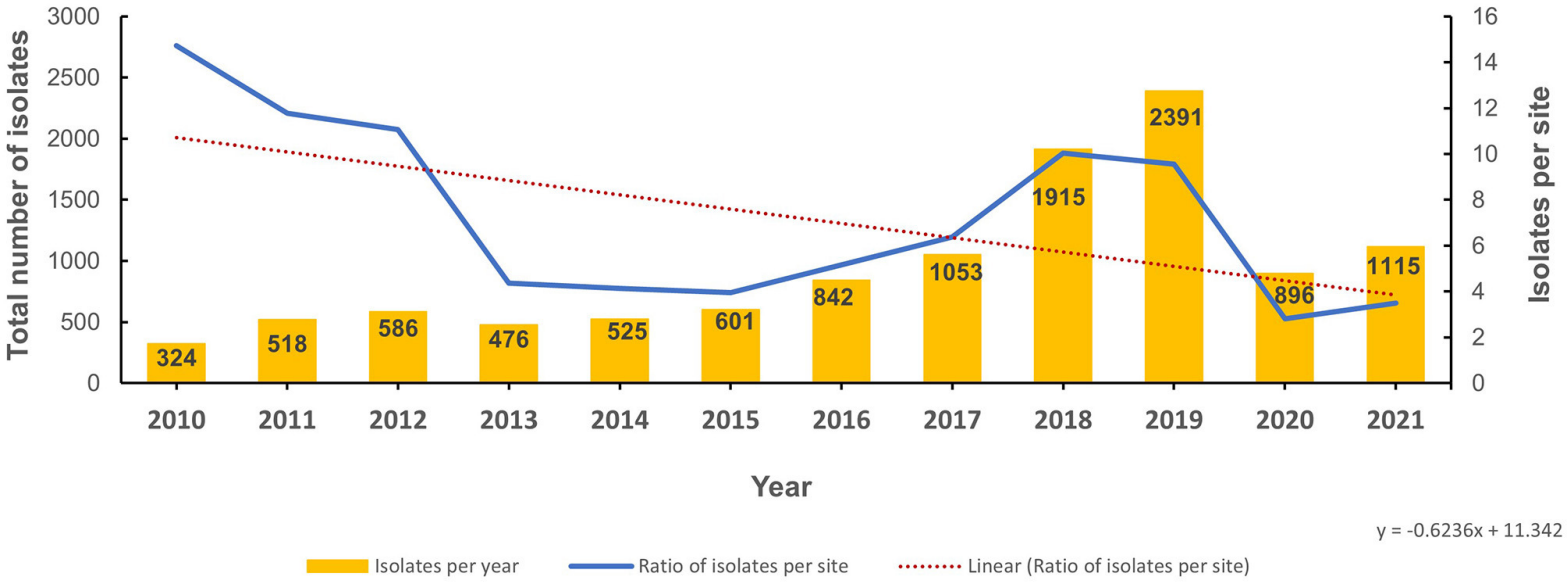
Number of *S. pneumoniae isolates* reported per year (2010–2021).

**Figure 3:**
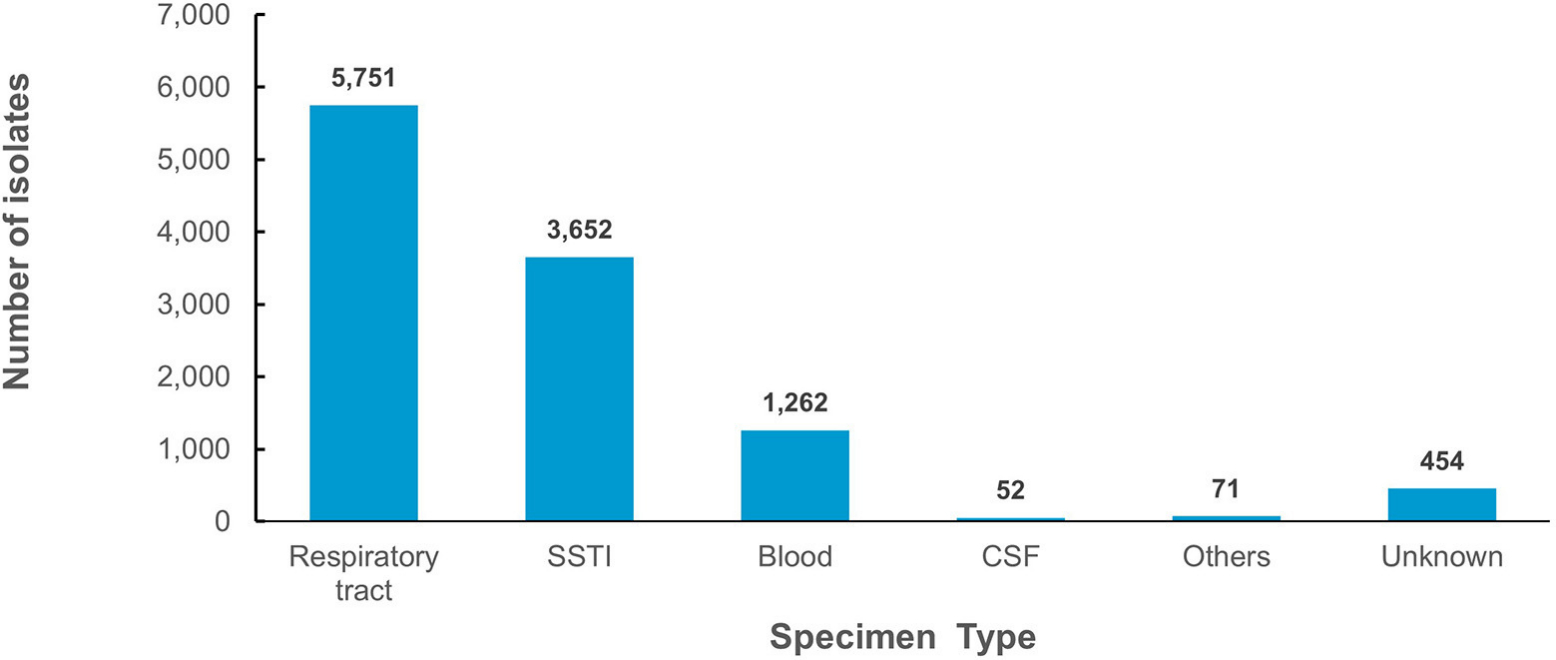
Distribution of *S. pneumoniae* islolates by specimen sources.

From 2010 to 2021, 11,242 non-duplicate isolates (representative of patients associated with *S. pneumoniae*) were reported, increasing from 324 in 2010 to 1,115 in 2021 (Figure 2). When normalized for the increased number of reporting sites per annum, after an initial decline between 2011 and 2014, the number of isolates per site increased between 2015 and 2018 with a plateau in 2019 (Figure 2). A sharp decline in reported *S. pneumoniae* isolates was observed in 2020 during the COVID-19 pandemic (Figure 2). The demographic distribution of the patients from whom isolates were obtained revealed a male preponderance with majority of patients being in the pediatric age group (Table 1). *S. pneumoniae* were predominantly isolated from respiratory tract specimens, which include nasopharyngeal swabs, sputum, bronchoalveolar lavage, tracheal aspirate and pleural fluid (*n/N* = 5,751/11,242; 51.2%). Isolates from nasopharyngeal swab specimens (indicative of colonization) represented 22.7% (*n/N* = 2,557/11,242) of all reported isolates and 44.5% (*n/N* = 2,557/5,751) of respiratory tract isolates. Figure 3 shows the distribution of specimen types where *S. pneumoniae* were isolated from. The isolation of *S. pneumoniae* in specimens from sterile sites which included blood, cerebrospinal, pleural and joint fluid was used as a marker of IPD. Up to 11.9% (*n/N* = 1,337/11,242) of all the *S. pneumoniae* isolates were from these sterile sites. Comparison of patients' demographics for IPD and non-IPD isolates revealed male preponderance in both groups with higher occurrence of hospitalization among IPD patients (51.1%), as compared to non-IPD patients (25.3%) (Table 2). There were more adult patients with IPD in contrast to the higher proportion of pediatric patients in the non-IPD group (Table 2). Patients with IPD were associated with a higher mortality rate (2.2%) as compared to patients with non-IPD (0.7%) (*p* < 0.001) (Table 2). Inpatients with IPD were associated with longer duration of hospitalization (median: 7 days), as compared to inpatients with non-IPD (median: 5 days). Changes in trend for IPD and non-IPD over time was only observed for nationality, with a decline in the percentage of Emirati nationals with IPD from 50.9% in 2010 to 33.3% in 2021, as well as those with non-IPD from 65.9% in 2010 to 40.4% in 2021.

**Table 1:** Demographic distribution of patients.

	**Number of patients (*****N*** = **11,242)**		**Percentage**
Gender	Male	5,597	49.8%
	Female	3,901	34.7%
	Unknown	1,744	15.5%
Age group	Pediatric	5,647	50.2%
	Adult	3,244	28.9%
	Unknown	2,351	20.9%
Nationality	Emirati	4,012	35.7%
	Non-Emirati	4,266	37.9%
	Unknown	2,964	26.4%
Patient location type	Outpatient	4,663	41.5
	Inpatient	3,124	27.8
	Emergency unit	1,430	12.7
	Intensive care unit	622	5.5
	Unknown	1,403	12.5

**Table 2:** Demographic distribution for invasive vs. non-invasive pneumococcal disease.

		**Patients with non-invasive pneumococcal disease *N* = 9,905 (88.1%)**	**Patients with invasive pneumococcal disease *N* = 1,337 (11.9%)**
Gender	Male	4,898 (49.4%)	699 (52.3%)
	Female	3,497 (35.3%)	404 (30.2%)
	Unknown	1,510 (15.2%)	234 (17.5%)
Age group	Pediatric	5,250 (53.0%)	397 (29.7%)
	Adult	2,637 (26.6%)	607 (45.4%)
	Unknown	2,018 (20.4%)	333 (24.9%)
Nationality	Emirati	3,553 (35.9%)	459 (34.3%)
	Non-Emirati	3,721 (37.6%)	545 (40.8%)
	Unknown	2,631 (26.6%)	333 (24.9%)
Patient location type	Outpatient	4,608 (46.5%)	55 (4.1%)
	Inpatient	2,506 (25.3%)	683 (51.1%)
	Emergency unit	1,129 (11.4%)	301 (22.5%)
	Intensive care unit	495 (5.0%)	127 (9.5%)
	Unknown	1,167 (11.8%)	171 (12.8%)
Clinical outcome	Discharged alive	2,787 (28.1%)	428 (32.0%)
	Discharged expired	70 (0.7%)	30 (2.2%)
	Unknown	7,048 (71.2%)	879 (65.7%)
Length of stay (inpatients)	Median inpatient LOS (days)	5	7

### 3.3 Antimicrobial susceptibility trends

**Figure 4:**
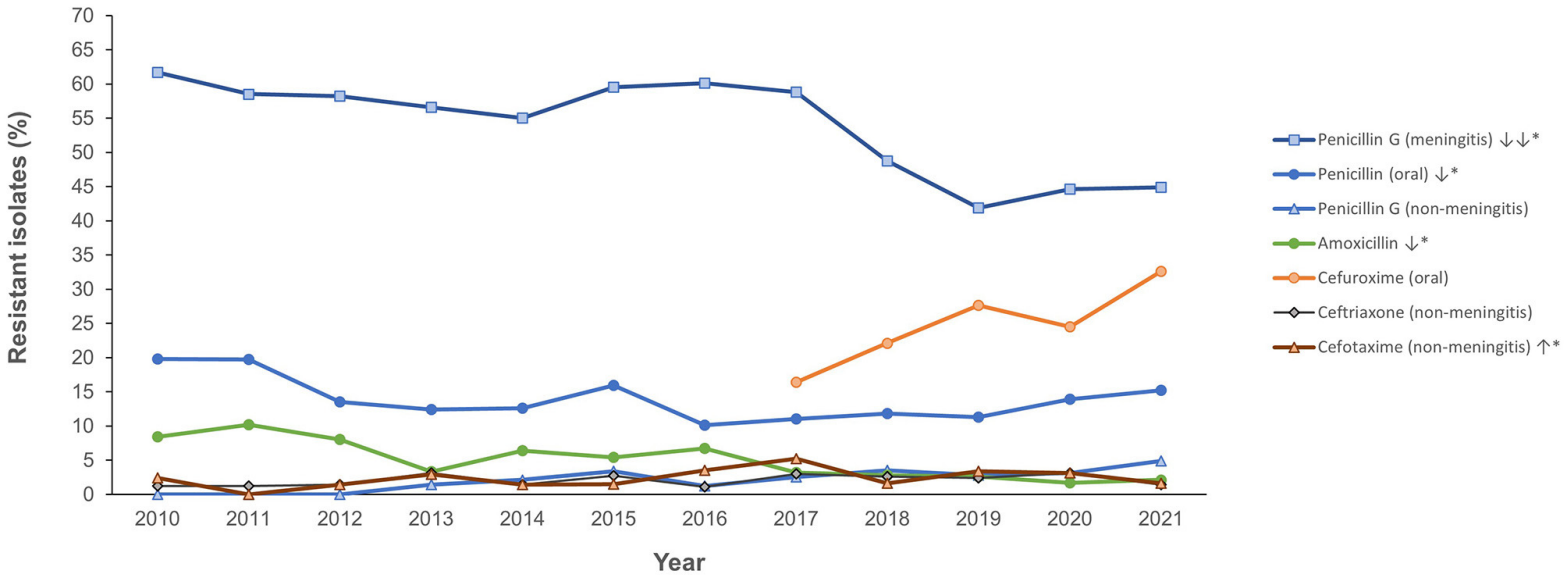
Resistance trend for beta lactam antibiotics (2010–2021). *Trend is statistically significant (*p* < 0.05).

**Figure 5:**
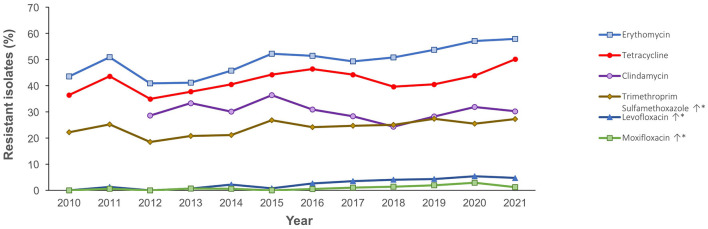
Resistance trend for other antibiotics (2010–2021). *Trend is statistically significant (*p* < 0.05).

**Figure 6:**
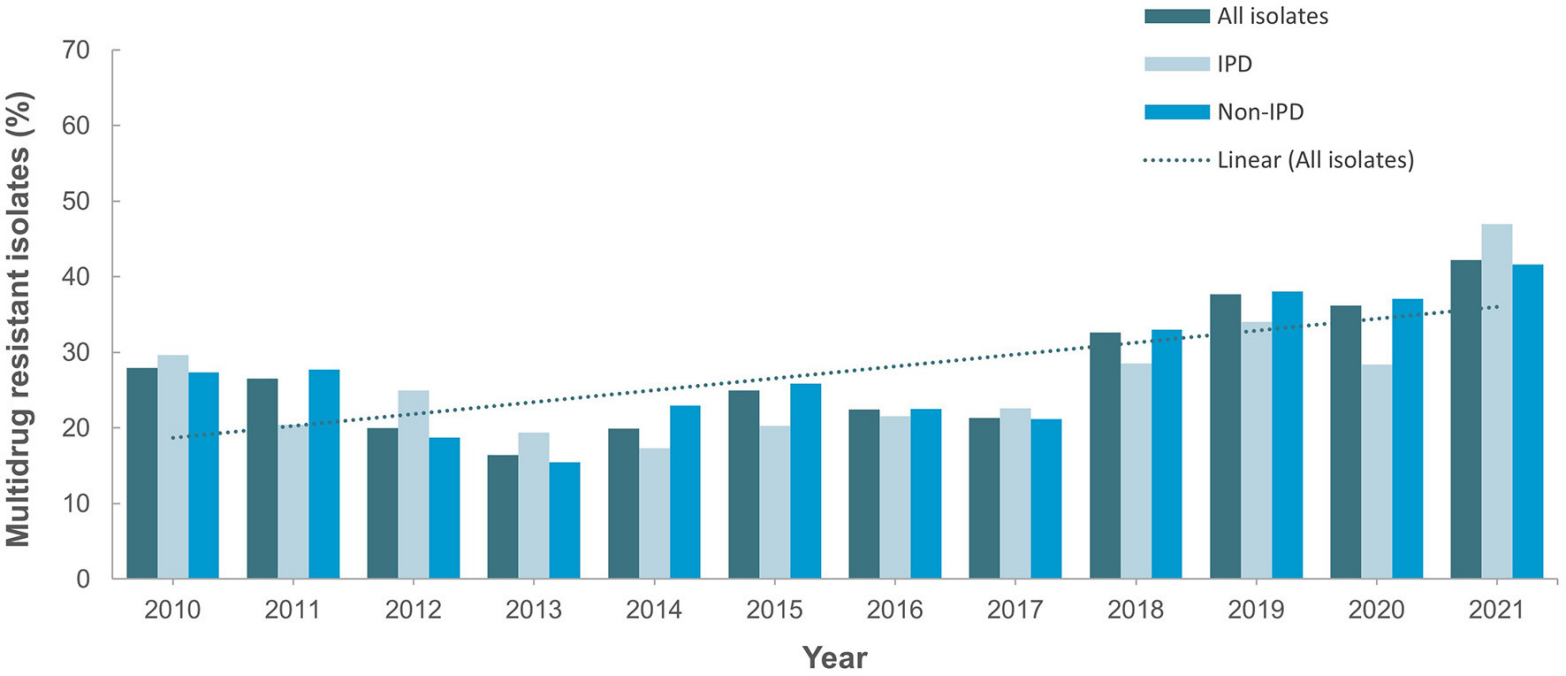
Trend for percentage multidrug-resistant isolates (2010–2021). Trend is statistically significant (*p* < 0.05).

Figure 4 shows the antimicrobial resistance trend for the beta lactam class of antibiotics. A statistically significant downward trend was observed for penicillin G at the non-meningitis and meningitis as well as oral penicillin breakpoints, and a similar trend was observed for amoxicillin (Figure 4). Despite the downward resistance trend, the proportion of *S. pneumoniae* isolates resistant to penicillin G at the meningitis breakpoint was over 45% which was much higher compared to other beta lactam antibiotics. For cefuroxime, although data was only available for 2017–2021, an upward trend in resistance was observed (Figure 4). For levofloxacin and moxifloxacin, although the proportion of resistant isolates was low across the study period (under 10%), there was an increasing trend which was statistically significant (Figure 5). Fluctuations were observed in the resistance trends for erythromycin, clindamycin, tetracycline and trimethoprim/sulfamethoxazole. However, the overall trends showed an upward trajectory which was statistically significant for trimethoprim/sulfamethoxazole (Figure 5). The proportion of multidrug resistant (MDR) isolates (resistance to 3 or more classes of antibiotics) increased from 16.4% (in 2013) to 42.2% in 2021 and this upward trend was statistically significant (*p* < 0.001; Figure 6). A comparison of IPD and non-IPD isolates did not reveal any differences between these two groups in the upward trend of MDR phenotype which was sustained at over 30% from 2018 to 2021 (Figure 6).

### 3.4 *S. pneumoniae* serotype distribution

None of the surveillance sites are currently conducting serotyping for *S. pneumoniae* and have not undertaken any serotyping in the preceding years (2010–2021). Hence no *S. pneumoniae* serotyping data was available for analysis. Handsearching of publicly available annual reports of health authorities for the period 2010–2020 did not yield data for *S. pneumoniae* serotype and burden of IPD.

## 4 Discussion

National surveillance programs are crucial for monitoring trends in pneumococcal disease and AMR patterns over time. We present the analysis of *S. pneumoniae* surveillance and AMR trends in the UAE over a period of 12 years (2010–2021). Our findings provide the first comprehensive epidemiological profile of pneumococcal disease in the UAE and the changing AMR trends which are of significance for clinical management. We demonstrate increased identification of *S. pneumoniae* in alignment with the increasing number of reporting sites over the data collection period. When normalized for number of study sites a sustained increasing trend of isolates per site observed from 2015 to 2019 which is consistent with global reports of rising pneumococcal disease burden (1, 23–25). The sharp decline observed in reported isolates in 2020 is suggestive of reduction in *S. pneumoniae* transmission during the first year of the COVID-19 pandemic. This could be attributable to the intense COVID-19 non-pharmacological interventions such as social distancing, masking and hand hygiene practices as well as the rapid change in healthcare-seeking behavior such as increased use of telemedicine and decreased hospital visits during the pandemic (26, 27). However, decreased testing and reporting during this period might have also resulted in the underestimation burden of pneumococcal disease.

Our findings show a male preponderance which may be a reflection of the demographic distribution of the UAE population but is also in keeping with reported literature from other studies (28, 29). The reasons for the male preponderance in pneumococcal disease are not fully understood hence further research is needed to elucidate the underlying factors as well as identify the potential implications for prevention and treatment strategies. The fact that most of our patients were in the pediatric age group is particularly relevant because nasopharyngeal carriage of *S. pneumoniae* is more prevalent in children compared to adults (30, 31). Indeed, nasopharyngeal isolates accounted for 22.7% of all isolates irrespective of specimen source and specifically for respiratory tract specimens, 44.4% of isolates were nasopharyngeal colonizers. Children are more likely to be colonized with *S. pneumoniae* due to their immature immune systems and increased exposure to respiratory pathogens and pneumococcal colonization contributes to the risk of IPD in children (24, 32). These findings underscore the need for targeted preventive measures to protect this vulnerable population (32). Vaccination against *S. pneumoniae* is particularly important in the pediatric population, as it has been associated with reduction in pneumococcal nasopharyngeal carriage leading to a reduced risk of IPD (33, 34).

Understanding the dynamics of pneumococcal disease and antimicrobial resistance is essential for developing robust intervention and treatment strategies. High levels of occurrence of nasopharyngeal carriage of *S. pneumoniae* can lead to the selection and spread of antibiotic resistant strains. Elevated levels of resistance to penicillin, macrolides and fluoroquinolone as well as the occurrence of multidrug resistance phenotype were reported in *S. pneumoniae* isolates in the UAE in a study carried out prior to the advent of the PCV vaccination program (16). In that report, of the 100 isolates identified between 2004 and 2006 only 57% were penicillin susceptible (16). Our findings which show high occurrence of penicillin resistant *S. pneumoniae* suggests that these isolates are now endemic in our setting. In contrast to the downward trend observed for beta-lactam antibiotics, resistance to quinolones, and macrolides showed an upward trend which is in keeping with findings from other countries in the region (35, 36). In Kuwait, the downward trend in penicillin resistance, has been shown to be associated with the introduction of the PCV and circulating serotypes (32, 35). The finding of similar trends of resistance for the two macrolide antibiotics aligns with previous report from the UAE which demonstrated high occurrence of level of cross-resistance between erythromycin and clindamycin (16). The ramifications of the increasing occurrence of *S. pneumoniae* MDR strains are immense including limitations on the effectiveness of antibiotics for the treatment of pneumococcal infections, which will further increase healthcare costs and the burden of disease. These findings underscore the need for continued surveillance and implementation of effective antibiotic stewardship programs to combat this growing AMR threat.

IPD and non-IPD differ in terms of their pathogenesis and clinical presentation. IPD is a severe and potentially life-threatening infection associated with invasion of sterile body sites while non-IPD is typically less severe and limited to non-sterile sites (37). The findings from this study represent the first insight into IPD burden in the UAE encapsulating the period post-commencement of the pneumococcal vaccination program. Expectedly, higher hospitalization with longer duration was observed for IPD patients. However, the high occurrence of MDR phenotype in both IPD and non-IPD isolates is a cause for concern as it suggests that emergence of resistant strains of *S. pneumoniae* is ongoing in our setting.

Our findings reveal the absence of data on *S. pneumoniae* serotypes circulating in our setting which is a limitation of the existing surveillance dataset. The emergence of non-vaccine serotypes and their increasing antimicrobial resistance is a reminder of the importance of ongoing surveillance to monitor changes in pneumococcal serotypes and their AMR patterns. Such data can inform the development of new and improved pneumococcal vaccines that provide coverage against a wider range of clinically relevant serotypes. We advocate for the urgent initiation of a national *S. pneumoniae* serotyping and genomic surveillance program as data from such initiative will be useful in guiding policy decisions for the introduction of new pneumococcal vaccines and vaccination schedules. In addition, such surveillance data will be useful for mapping genomic changes associated with serotype switching from vaccine pressure, as well as provide early warnings for emergence of resistance and the spread of global clones. The occurrence of a high percentage of missing data and information about specific population risk groups are limitations observed in this national AMR surveillance dataset. This highlights the need for continued provision of training to personnel at participating sites as well as expansion of the clinical parameters included in the dataset.

## 5 Conclusion

Our analysis of the national *S. pneumoniae* AMR surveillance data provides insights into the evolving patterns of pneumococcal disease and antimicrobial resistance in the UAE. The findings highlight the need for the introduction of a *S. pneumoniae* serotype surveillance program to guide the pneumococcal vaccination program, as well as continued AMR monitoring and targeted intervention measures to address the growing threat of antibiotic resistance.

## Data availability statement

The datasets presented in this article are not readily available because the National AMR Surveillance database managed by the UAE Ministry of Health and Prevention (MOHAP) contains confidential health information. Requests to access the datasets should be directed to https://mohap.gov.ae/.

## Ethics statement

Ethical approval for this study was provided by the Ministry of Health and Prevention Research Ethics Committee (MOHAP/DXB-REC/D.D.D/No.131/2021; MOHAP/DXB-REC/J.J.J./No. 86/2023), Dubai Scientific Research Ethics Committee (DSREC-GL17-2023), and Abu Dhabi Health Research and Technology Ethics Committee (DOH/ZHCD/2023/1316). The studies were conducted in accordance with the local legislation and institutional requirements. Written informed consent for participation was not required from the participants or the participants' legal guardians/next of kin in accordance with the national legislation and institutional requirements.

## Author contributions

Conceptualization, data interpretation, and manuscript review and editing: AS, JT, NA, GM, CA, and DE. Data collection: AS, JT, NA, GM, CA, DE, and The UAE AMR surveillance consortium. Formal analysis and manuscript preparation: AS and JT. All authors have read and agreed to the published version of the manuscript.

## Footnotes

^1^WHONET. The laboratory database software. https://whonet.org/.

## Appendix

**Table A1:** The UAE AMR Surveillance Consortium.

**Nr**.	**Name**	**Institution**
1	Ahmed Elhag Ahmed	UAE University, College of Medicine and Health Sciences, Al Ain
2	Ahmed F. Yousef	Department of Biology, Center for Membranes and Advanced Water Technology, Khalifa University, Abu Dhabi
3	Amna AlBlooshi	Purelab, Al Ain
4	Dr. Adnan Alatoom	Sheikh Shakhbout Medical City (SSMC), Abu Dhabi
5	Dr. Ahmed Abdulkareem Al Hammadi	Tawam Hospital, Al Ain
6	Dr. Alaa MM Enshasy	Dubai Health Authority, Dubai
7	Dr. Amal Mubarak Madhi	Abu Dhabi Public Health Center, Abu Dhabi
8	Dr. Anju Nabi	Dubai Academic Health Corporation (DAHC), Dubai
9	Dr. Anup Shashikant Poddar	Al Sharq Hospital, Fujairah
10	Dr. Arun Kumar Jha	Danat Al Emarat Hospital, Abu Dhabi
11	Dr. Ayesha Abdulla Al Marzooqi	Abu Dhabi Public Health Center, Abu Dhabi
12	Dr. Bashir Aden	Khalifa University, Abu Dhabi
13	Dr. Deeba Jafri	Purelab, Sheikh Khalifa Medical City, Ajman
14	Dr. Duckjin Hong	Sheikh Khalifa Specialty Hospital (SKSH) RAK
15	Dr. Farah Ibrahim Al-Marzooq	United Arab Emirates University, Al Ain
16	Dr. Fatima Al Dhaheri	United Arab Emirates University, Al Ain
17	Dr. Ghada Abdel Wahab	Abu Dhabi Agriculture and Food Safety Authority, Abu Dhabi
18	Dr. Ghalia Abdul Khader Khoder	University of Sharjah, Sharjah
19	Dr. Gitanjali Avishkar Patil	NMC Specialty Hospital, Abu Dhabi
20	Dr. Hafiz Ahmad	RAK Hospital, Ras Al Khaimah
21	Dr. Hazim Khalifa	Department of Veterinary Medicine, UAE University, Al Ain
22	Dr. Husein Alzabi	Sheikh Khalifa General Hospital, Um al Quwain
23	Dr. Ibrahim Alsayed Mustafa Alhashami	Purelab, Al Qassimi Hospital, Sharjah
24	Dr. Irfaan Akthar	Mediclinic City Hospital, Dubai
25	Dr. Jens Thomsen	Abu Dhabi Public Health Center, Abu Dhabi
26	Dr. John Stelling	WHONET, Boston, USA
27	Dr. Kavita Diddi	Prime Hospital, Dubai
28	Dr. Krishnaprasad Ramabhadran	Burjeel Hospital, Abu Dhabi
29	Dr. Laila Al Dabal	Dubai Academic Health Corporation (DAHC, Dubai)
30	Dr. Madikay Senghore	Khalifa University, Abu Dhabi
31	Dr. Manal Abdel Fattah Ahmed	PureLab, Ras Al Khaimah
32	Dr. Maya Habous	Rashid Hospital, Dubai Academic Health Corporation, Dubai
33	Dr. Moeena Zain	American Hospital Dubai
34	Dr. Monika Maheshwari	Al Zahra Hospital, Dubai
35	Dr. Monika Maheshwari	Medeor 24 × 7 Hospital, Dubai
36	Dr. Mubarak Saif Alfaresi	Zayed Military Hospital, Abu Dhabi
37	Dr. Mushtaq Khan	United Arab Emirates University, Al Ain
38	Dr. Najiba Abdulrazzaq	Al Kuwait Hospital, Emirates Health Services Establishment, Dubai
39	Dr. Nehad Nabeel Al Shirawi	Al Fujairah Hospital
40	Dr. Nesrin Helmy	Mediclinic Al Noor Hospital - Khalifa Street, Abu Dhabi
41	Dr. Prashant Nasa	NMC Specialty Hospital Al Nahda, Dubai
42	Dr. Rajeshwari T. A. Patil	Burjeel Medical City, Abu Dhabi
43	Dr. Ratna A. Kurahatti	NMC Royal Hospital Khalifa City A, Abu Dhabi
44	Dr. Riyaz Amirali Husain	Dubai Hospital, Dubai Academic Health Corporation, Dubai
45	Dr. Robert Lodu Serafino Wani Swaka	Sheikh Shakhbout Medical City, Abu Dhabi
46	Dr. Savitha Mudalagiriyappa	University Hospital Sharjah, Sharjah
47	Dr. Seema Oommen	Burjeel Medical City, Abu Dhabi
48	Dr. Shaikha Ghannam Alkaabi	Abu Dhabi Public Health Center, Abu Dhabi
49	Dr. Simantini Jog	Fakeeh University Hospital, Dubai
50	Dr. Simantini Jog	King's College Hospital London Dubai Hills, Dubai
51	Dr. Siobhan O‘Sullivan	Khalifa University, Abu Dhabi
52	Dr. Somansu Basu	NMC Specialty Hospital, Al Ain
53	Dr. Yassir Mohammed Eltahir Ali	Animal Wealth Sector, Abu Dhabi Agriculture and Food Safety Authority, Abu Dhabi
54	Dr. Yousuf Mustafa Naqvi	Department of Health Abu Dhabi (DoH), Abu Dhabi
55	Dr. Zulfa Omar Al Deesi	Latifa Maternity & Pediatric Hospital, Dubai
56	Emmanuel Fru Nsutebu	Sheikh Shakhbout Medical City, Abu Dhabi
57	Fouzia Jabeen	Purelab, Sheikh Khalifa Hospital, Abu Dhabi
58	Francis Amirtharaj Selvaraj	Sheikh Khalifa Medical City (SKMC), Abu Dhabi
59	Hadayatullah Ghulam Muhammad	Emirates International Hospital, Al Ain
60	Imene Lazreg	University of Sharjah, Sharjah
61	Kaltham Ali Kayaf	Ministry of Climate Change & Environment (MOCCAE), Dubai
62	Laura Thomsen	University of Freiburg, Germany
63	Leili Chamani-Tabriz	Clemenceau Medical Center, Dubai
64	Pamela Fares Mrad	Abu Dhabi Public Health Center (ADPHC), Abu Dhabi
65	Pascal Frey	Berne University Hospital, Berne, Switzerland
66	Prof. Abiola Senok	College of Medicine, Mohammed Bin Rashid University of Medicine and Health Sciences, Dubai
67	Prof. Agnes-Sonnevend-Pal	University of Pécs, Pécs, Hungary
68	Prof. Andreas Podbielski	University Hospital Rostock, Rostock, Germany
69	Prof. Carole Ayoub Moubareck	College of Natural and Health Sciences, Zayed University, Dubai
70	Prof. Dean Everett	Department of Pathology and Infectious Diseases, College of Medicine, Khalifa University, Abu Dhabi
71	Prof. Godfred A. Menezes	Department of Medical Microbiology and Immunology, RAK Medical and Health Sciences University, Ras Al Khaimah
72	Prof. Hala Ahmed Fouad Ismail	PureLab, Al Qassimi Hospital, Sharjah
73	Prof. Mohamud M. Sheek-Hussein	United Arab Emirates University, Al Ain
74	Prof. Peter Nyasulu	Department of Global Health, Faculty of Medicine and Health Sciences, Stellenbosch University, South Africa
75	Prof. Sameh Soliman	University of Sharjah, Sharjah
76	Prof. Tibor Pal	University of Pécs, Pécs, Hungary
77	Rania El Lababidi	Dept. of Pharmacy Services, Cleveland Clinic Abu Dhabi
78	Saeed Hussein	Erada Center for Treatment and Rehabilitation, Dubai
79	Stefan Weber	Purelab, Abu Dhabi
80	Sura Khamees Majeed	Al Gharbia Hospitals - Madinat Zayed Hospital
81	Syed Irfan Hussein Rizvi	Mediclinic City Hospital, Dubai
82	Timothy Anthony Collyns	Tawam Hospital, Al Ain
83	Zahir Osman Babiker	Sheikh Shakhbout Medical City (SSMC), Abu Dhabi
